# Health needs assessment for the double burden of malnutrition: a community-based study on nutrition facilitators and barriers in rural Tanzania

**DOI:** 10.1017/S1368980023001568

**Published:** 2023-11

**Authors:** Victoria von Salmuth, Lieke Buijs, Bwire Chirangi, Anita CE Vreugdenhil, Onno CP van Schayck

**Affiliations:** 1 Department of Family Medicine, CAPHRI, Maastricht University, Maastricht, The Netherlands; 2 Faculty of Health, Medicine and Life Sciences, Maastricht University, Maastricht, The Netherlands; 3 Shirati KMT District Hospital, Rorya, Mara, Shirati, Tanzania; 4 Department of Paediatrics, NUTRIM School of Nutrition and Translational Research in Metabolism, Maastricht University Medical Centre, Maastricht, The Netherlands

**Keywords:** Malnutrition, Maternal health, Child health, Double burden of malnutrition, Tanzania

## Abstract

**Objective:**

The aim of this study is to explore nutrition-related health needs, the perceptions and beliefs regarding the double burden of malnutrition, as well as barriers and facilitators in accessing nutritious food among the local population in rural Tanzania.

**Design:**

A qualitative study design using semi-structured individual interviews and focus-group discussions (FGD) was used. Basic socio-demographic information was obtained from all participants.

**Setting:**

The study was conducted in four villages within the catchment area of the Shirati KMT Hospital in Rorya district, in north-western Tanzania.

**Participants:**

Men and women in the reproductive age as well as Community Health Workers (CHW) were included.

**Results:**

In total, we performed fourteen interviews (*N* 41), consisting of four FGD, one dual and nine individual interviews. The three most significant topics that were identified are the large knowledge gap concerning overweight and obesity as a health problem, changing weather patterns and its implications on food supply and the socio-cultural drivers including gender roles and household dynamics.

**Conclusion:**

Environmental and socio-cultural factors play a crucial role in the determinants for DBM, which underlines the importance of understanding the local context and the nutrition practices and beliefs of the communities. Future nutritional interventions should aim towards more inclusion of men in project implementation as well as support of women empowerment. CHW could play a key role in facilitating some of the suggested interventions, including nutritional counselling and increasing awareness on the drivers of the double burden of malnutrition.

Maternal and child malnutrition constitutes a major global health burden in low- and middle-income countries^(1–3)^. While the number of stunted children under the age of five has decreased globally over the last three decades, the prevalence of maternal and childhood obesity is further rising, contributing significantly to the morbidity and mortality rates on a global scale with the highest burden prevailing in South-central Asia and Sub-Saharan Africa (SSA)^(2)^. Despite the progress made on counteracting the issue of undernutrition in Tanzania, chronic malnutrition remains one of the leading contributors to child mortality, with an estimated stunting rate of 32% in children below 5 years^(4,5)^. At the same time, the issue of overweight and obesity has emerged as a new public health challenge in Tanzania, especially among women in the reproductive age, with an increase in prevalence of 17 % in the period from 1991 to 2016^(6,7)^.

The co-existence of different types of malnutrition and diet-related non-communicable diseases within individuals, households and communities is referred to as the double burden of malnutrition (DBM)^(8)^. While inadequate intake of nutritious food, lack of dietary diversity and infectious diseases are considered immediate causes for malnutrition, underlining challenges such as widespread poverty, poor living conditions and lack of access to healthcare significantly contribute to the DBM^(2,9,10)^. In addition, the impact of climate change related changes in weather patterns, and the secondary effects of the COVID-19 containment measures further increased the risk of malnutrition among children and women in the reproductive age in many countries in SSA^(11–14)^.

Maternal nutritional status has been widely recognised as crucial factor for the health, growth and development of infants, especially in the period of pre-conception, pregnancy and the first two years of a child’s life^(15–17)^. Poor maternal nutritional status is associated with adverse health and reproductive outcomes in women. In children, it can have a direct impact on physical growth and cognitive development, which can lead to lifelong impairments^(5,7)^. Other maternal factors such age, literacy, employment and empowerment significantly influence childhood as well as maternal health and nutritional status^(18)^. Traditionally, men are considered the provider and the controller of the financial resources in many Tanzanian households, whereas the purchase and preparation of food as well as child feeding is often regarded as sole responsibility of the women. Existing literature shows that improving maternal decision-making capacity and empowerment of women might positively improve household well-being, including child nutrition^(19)^. On the other hand, there has also been a shift in recent years towards more involvement of men in nutritional responsibilities on household level in East Africa, providing new opportunities for future interventions^(20,21)^.

Treatment programmes in SSA, including Tanzania, have predominantly focused on tackling hunger and undernutrition. The recently launched second Tanzanian National Multi-Sectoral Nutrition Action Plan (2021–2026), however, recognises the issue of a triple burden of malnutrition, including undernutrition/stunting, micronutrient deficiency and overweight/ obesity, and strives towards a multi-sectoral approach to tackle this issue^(22)^. This is in line with the policy developments by the WHO, that have been observed from 2017 onwards, outlining the common drivers of DBM and possible interventions on how to address them^(8)^. So-called double-duty actions are aiming at tackling multiple forms of malnutrition simultaneously and have been recommended by international bodies to tackle the DBM^(8,23)^. These recommendations and plans, however, have largely not been taken up at a national and regional level yet^(24)^. Very limited data are available on current food security or nutrition programmes in the study area in Rorya district, Tanzania. To our knowledge, there are several School Feeding Programs in place in the region that focus on provision of food in primary schools, health education and capacity building^(25)^. We are not aware of any other programmes that are focusing specifically on the DBM or triple burden of malnutrition in the study area.

A key step towards future intervention programmes and implementation of the policies in Tanzania is to develop a better understanding of the views of local communities, their experiences in nutritional practices, beliefs and preferences when it comes to a healthy diet. Community Health Workers (CHW), members of the community who provide basic medical care or public health counselling particular in rural areas, could play a major role in enhancing the development and implementation of nutrition intervention in rural Tanzania^(26)^. Therefore, this research project aims at increasing insight in local perceptions and beliefs concerning the DBM, discussing drivers of malnutrition and exploring current health needs in Rorya district in rural Tanzania.

## Methods

A qualitative study, using semi-structured individual interviews and Focus Group Discussions (FGD), was performed in four rural villages to explore nutrition-related health needs priorities of local population in the Rorya district, Tanzania. The interviews took place in the period of October 2019 to December 2019.

### Study setting

The Rorya district is situated in north-western Tanzania on the shore of Lake Victoria and adjacent to the Kenyan border. The largest town of the district, Shirati, populates approximately 100 000 people, with almost half of them being children under the age of 14^(27)^. This town consists of widespread rural villages and situates the largest medical care facility of the Rorya district, the Shirati KMT Hospital.

The study was conducted in four villages within the catchment area of the Shirati KMT Hospital, namely Masike, Nyamagaro, Panyakoo and Sota. The research locations were purposively selected based on the representation of all varieties of cultures, road access, distance to the lake as well as registered cases of malnutrition among children under the age of five. Current data on the prevalence of overweight and obesity in the respective communities were not available.

### Participants

Men and women in the reproductive age (15–49 years) were included in the study, to gain insight in the different health needs and nutrition-related gender roles. Female participants were eligible when they were pregnant or had at least one child under the age of 5. In addition, one CHW from every research location was included in the study.

### Recruitment

Eligible community members who were already present at the health facility of the research locations were recruited in collaboration with local nurses and CHW. This sampling technique was used because of its feasibility in a rural area with minimal communication resources. Participation of all women, men and CHW was voluntary. Women in the reproductive age participated in FGD, whereas men and CHW participated in individual interviews. Participants were provided with an elaborate verbal and written explanation of the study purposes and explicitly assured of their anonymity before they were enrolled in the research.

### Data collection

Interviews took place in or around the local health facility of the respective villages. FDG were conducted with women and men separated from each other in order to increase participants’ comfort to discuss sensitive topics. FGD lasted 60–90 min and individual interviews lasted 30–60 min. Each interview was conducted by the same research team. One experienced, female translator, who spoke English, Kiswahili and the local language Luo, performed direct translations during all interviews. In total, we performed fourteen interviews, of which four FGD with a total of thirty women, one dual and nine individual interviews with men (*n* 7) and CHW (*n* 4).

Interviews were semi-structured, guided by pre-developed opening questions. The interview guide for male and female participants was partly based on the common DBM drivers established by the WHO^(8)^ and has been piloted beforehand. Small adaptations had been made according to the results of this pilot. The interview guide was back translated to ensure coherent translation. It consisted of nutrition-related questions on accessibility, perceptions, socio-cultural context, economic, environment, health, maternity and education and concluded with an overall health needs assessment. The interview guide for CHW was developed separately and contained questions focused on their backgrounds, knowledge, activities and challenges. Interviews with women, men and CHW were performed until data saturation was reached. Interview observers were present during the interviews, consisting of one international researcher and one local staff member. All interviews were recorded with a digital voice recorder and later transcribed verbatim. Participants’ names were anonymised by the use of descriptive references. Basic socio-demographic information was obtained from all participants prior to the interviews.

### Data analysis

Content analysis of the interview transcription data was performed by the primary researcher using the qualitative data analysis software QRS Nvivo 12. Thematic analysis was performed according to the six-step approach, including familiarisation with the data, generating of the initial codes, identification of themes, defining and naming theme and finally composing the written analysis^(28)^.

The code scheme was shared and discussed with the interview observers and an independent data analyst. Themes include the local perception of healthy diets and malnutrition, barriers and facilitators of malnutrition in different environments, such as food, economic, health and socio-cultural environments and possible interventions to tackle the DBM.

## Results

In total, we performed fourteen interviews, of which four FGD with women (*n* 30), one dual and nine individual interviews with men (*n* 7) and CHW (*n* 4). Age, gender and socio-demographic characteristics of respondents are summarised in Table 1.

**Table 1:** Socio-demographic characteristics of participants

	Total (*n* 41)		Women (*n* 30)		Men (*n* 7)		CHW (*n* 4)	
Variables	Mean	sd	Mean	sd	Mean	sd	Mean	sd
Age	31·9	−10·1	28·4	−7·3	35·7	−7·3	51·8	−6·5
	*n*	%	*n*	%	*n*	%	*n*	%
Gender								
Male	9	22	0	0	7	100	2	50
Female	32	78	30	100	0	0	2	50
Location								
Masike	11	27	8	27	2	29	1	25
Nyamagaro	10	24	7	23	2	29	1	25
Panyakoo	10	24	7	23	2	29	1	25
Sota	10	24	8	27	1	14	1	25
Household size								
1–5 persons	6	15	4	13	1	14	1	25
6–10 persons	34	83	25	83	6	86	3	75
> 10 persons	1	2	1	3	0	0	0	0
Relationship status								
Single	1	2	1	3	0	0	0	0
In an unmarried relationship	2	5	1	3	1	14	0	0
Monogamous marriage	25	61	19	63	5	71	1	25
Polygamous marriage	11	27	7	23	1	14	3	75
Divorced	1	2	1	3	0	0	0	0
Widowed	1	2	1	3	0	0	0	0
Children								
None	2	5	1	3	1	14	0	0
1–3 children	10	24	9	30	1	14	0	0
4–6 children	16	39	13	43	2	29	1	25
7–9 children	13	32	7	23	3	43	3	75
Pregnant women	3	7	3	10	n.a.	n.a.	n.a.	n.a.
Finished level of education								
None	2	5	2	7	0	0	0	0
Primary education	33	80	24	80	6	86	3	75
Secondary education	5	12	3	10	1	14	1	25
Unknown	1	2	1	3	0	0	0	0
Occupation								
Unemployed	9	22	9	30	0	0	0	0
Student	1	2	1	3	0	0	0	0
Self-employed	30	73	19	63	7	100	4	100
Unknown	1	2	1	3	0	0	0	0

CHW, Community Health Worker.

In total we had forty-one participants, with the majority 78 %, being women, and 22 % men. The mean age of the participants was 31·9 years. Women on average were younger (mean sd 38·4 years), compared with men (mean sd 35·7 years) and CHW (mean sd 51·8 years). The average household size was 6–10 persons, with the majority having between four to nine children. About 80 % of all participants finished primary education, without considerable differences between men and women, whereas only 12 % finished secondary school. The majority of participants reported to be self-employed, 22 % of all participants reported that they were unemployed, all of them being women.

### Theme I – current nutrition perceptions

#### Perceptions of a healthy diet

In general, participants were aware that a balanced diet consists of a mixture of different types of food. Acquiring knowledge on nutrition-related issues was considered a pre-requisite for tackling different causes of malnutrition, as a 23-year-old female from Masike affirmed; ‘It is only because of money, but I am aware, I know of what a balanced diet is. So if I have the money I can always afford to do that [have a balanced diet]’. However, a few participants claimed that one certain type of food or drink is involved in making a diet healthy, as a 31-year-old man from Masike said; ‘You must have ugali, for it to be a balanced diet’. Ugali is a stiff porridge made of maize, cassava or millet flour.

Women appeared to be aware of the general recommendation concerning exclusive breastfeeding in infants up to 6 months. In addition, some women stated that they are adapting their diet during pregnancy and during the breastfeeding period, by either eating more meals per day or by having a greater variety of food. A 26-year-old female participant from Sota explained that according to her knowledge; ‘In addition to your meals, you should add like vegetables and fruits’. However, most men and some women were less familiar with adequate maternal nutrition practices, as recommended by the WHO^(29)^. Respondents frequently stated that a pregnant woman should eat according to her preferences, which could mean either eating other food types, larger quantities, or also smaller portions.

#### Perceptions of malnutrition

The lack of a balanced diet was generally associated with undernutrition and weight loss. Most participants were able to name some physical signs of acute malnutrition in children, such as thinness of the cheeks and limbs, distended abdomen and brown hair. Malnutrition in adults, however, was not perceived as a common problem, except for adults with chronic infectious diseases. Many participants stated that they have difficulties detecting weight loss or weight gain, both in themselves and in their children. They reported that unless they go to the hospital or dispensary to measure their weight, weight changes would remain unnoticed.

Many interviewees reported that the number of people with overweight or obesity was low in their respective communities; however, some admitted that they did not know how to define overweight. Interviewees frequently stated that overweight is identified according to a person’s visible body size, his sedentary lifestyle or based on a weight cut-off point of for example 100 kg without considering the person’s height. Respondents associated weight gain to a lack of stress, living in urban areas and increasing income, as a 32-year-old male participant from Panyakoo declared; ‘[…] there are times I have to go to a city, and then I have to sit and eat and no any hard work. So when I go there, I experience weight gain’. A few participants were aware that non-communicable diseases like hypertension and diabetes are paramount consequences of overweight, whereas some CHW-related high blood pressure particularly to acute stress.

### Theme II – facilitators and barriers for access to adequate nutrition

#### Food environment

The main source of food for in Rorya district derives from farming and fishing activities. Most respondents grow cassava on their farms, which they use to make ugali. Ugali is a common basis in traditional Tanzanian meals, often combined with small sardine-like fish or green leafy vegetables^(30)^. A number of female participants in Masike mentioned that they have access to varying types of green leafy vegetables in the surroundings, as a 25-year-old woman explained; ‘They [the vegetables] just grow in the farm, we do not plant them ourselves’. Other types of food that cannot be collected from the farms or the lake, they have to buy at the market. Food types that are less commonly consumed including sweet potatoes, bigger fish, meat, rice, beans, milk or eggs.

Most participants are dependent on sufficient local irrigation of their farmland during the rainy season. Drought and the absence of an irrigation system impacts the availability of food both on their farms and at the market, as food prices rise significantly during the dry season. On top of that, respondents from different villages affirmed that rain patterns changed throughout the last couple of years, which is increasingly contributing to food insecurity in the region.

‘In the olden days there used to be much rain, unlike now. So, when there was much rain, there was much food. But now there is less rain, so there is less food’. (28-year-old woman from Nyamagaro)

Rainy season is associated with an increased work demand on the farms, which could restrain the farmers time for other income-generating activities. Furthermore, it was stated that unseasonal precipitation and unexpected heavy rains can wash away crops, which in turn negatively affects the harvest.

Physical distance to markets is generally not perceived as a barrier for access to food; however, a 34-year-old woman in Masike explained that sometimes she has to travel relatively far to purchase fruits for consumption. Other environmental barriers that have been reported to affect people’s access to food include an increased use of illegal fish toxines in Lake Victoria and a lack of access to high quality planting seeds.

#### Economic environment

Income-generating activities that enable the purchase of food products at the market include selling farming and fishing products, burning charcoal, riding a motor taxi and being a carpenter. Most respondents explained that the harvests from their farms are primarily used for personal consumption. Seldomly, a small share of the farming products is being sold on the market in order to provide income for basic needs like soap. Thus, owning a small piece land is associated with a harvest yield that is only sufficient to supply the family’s needs and would not allow for market sales.

Participants stated that the lack of sufficient starting capital to set up a business is another barrier to earning a sufficient income. Participants specified that diseases or physical disabilities critically influence their income, as they are mostly dependent on income-generating activities that require physical activity and strength.

A lack of income was linked to the consumption of fewer meals and/or a meal that consists of a very limited variety of foods.

‘If I have enough money, first of all what I have to buy is the flour, it is either maize. And then I will think of maybe fish or small fish and some fruits, like banana, avocado. But if I have the least money, I always think of having the flour and then go all over the surroundings and look for the vegetables’. (25-year-old woman from Masike)

Several women also mentioned that financial constraints result in a decreased compliance to recommended maternal nutrition practices and early introduction of complementary feeding:

‘Always you have to breastfeed up to 6 months, but it depends with how you get your food. So, if you go hungry most of the time, then as early as you feel that your child cannot get enough milk from you, you just give them anything that is there. If it is uji [porridge], if it is potatoes, whatever, you just give. Because you not have much to have more milk production to make the baby feel satisfied’. (30-year-old woman from Panyakoo)

#### Health environment

Participants stated that they have not been taught on nutrition-related issues before. Education projects offered by non-governmental organisations and governmental organisations are traditionally focused on topics such as family planning, water and sanitation and health seeking behaviour, rather than on nutrition. Some CHW explained that they regularly provide basic nutrition counselling to patients at the antenatal clinics, in particular to mothers with malnourished children or to clients with HIV/AIDS. This was confirmed by a number of female participants. As a result, villagers who do not visit the local health facility rarely receive any nutrition education.

‘It is only women who get the [nutrition] education because the women are the ones who come to the antenatal clinic, they are the ones who bring the babies for vaccination. And when they work door to door, rarely do they find the men at home’. (37-year-old woman from Masike)

The facilitation of health care activities by CHW in the communities fluctuates, as projects come and go. Most of the activities depend on supplies and training that are part of temporary projects organised by various non-governmental organisations and government incentives. The absence of medicines and medical equipment at the dispensaries is commonly reported as barrier for access to health care. A CHW from Masike explained; ‘There is only advice here and leave. We do not have any other things to give’.

#### Socio-cultural environment

Most male participants stated that the decision making in relation to food is done by both men and women together. Female participants, however, disclosed that their husbands generally decide what food is eaten by the family on a daily basis because they are the ones who earn the money.

‘I think it is the man who decides on whatever to be bought. Because you can think: “Ok, I need to buy this”. But at the time you get the food on the table, the husband might say: “No, I do not want that food. Who told you to buy that?” So it brings some misunderstanding. So it is always better, if you want to buy food, you must ask him first: “Ok, what am I going to buy?”’ (35-year-old woman from Panyakoo)

Male interviewees affirmed that they share all food on the table with every family member, regardless of the amount of food or type of food available. The majority of participants expressed that they primarily assure that their children have enough to eat, before they eat themselves. However, many women admitted that ‘the best portion and the biggest and the fattest portion is always left for the men. Because they are the ones who always bring out the money’, as was reported by a 36-year-old woman in Sota. In some families, prioritisation of the husband even results in an inadequate diet for children:

‘If you find such type of husband then always the children go with only the soup and the ugali, and they will have to leave the food for the husband’. (29-year-old woman from Masike)

Lack of support by the husband also appeared to be a barrier in adequate access to maternal nutrition. Some women declared that they rely heavily on their husbands’ understanding and support concerning their food intake during pregnancy and breast-feeding.

### Theme III – possible nutrition interventions

A frequently stated intervention opportunity is tackling the food insecurity problem by providing food. One suggestion was to provide food to the poorest families in the community, or to those who are already suffering from malnutrition.

‘If you can give the support on the provision of food, you can have like some representatives who can go and see in the community nearby where there are children who have some problems like malnutrition, and then provide it to them’. (46-year-old man from Nyamagaro)

Most CHW suggested that a continuous provision of therapeutic foods and supplements for acutely malnourished children would tackle the main issue of undernutrition. Some CHW stated that they are willing to screen children for malnutrition and also do a follow-up on them, but only if treatment is available for diagnosed patients. The CHW from Panyakoo suggested that the Kenyan health care system could be taken as an example:

‘You should have some nutritional supplements, which are available in the dispensary here. Because most women always go to the nearby country, because there, if you have malnourished children, you get the nutritious food’. (CHW from Panyakoo)

The CHW from Nyamagaro expressed concerns that the problem of increasing drought needs to be tackled by a different way of farming; ‘if we move to the side of the lake and have some irrigation machines and whatever, then that can be successful’. The need for a constant water supply was also mentioned by a 38-year-old man from Masike; ‘Because we do not have the irrigation system, we cannot be sure of getting enough food or enough food to sell or to give to the family’.

Most of the intervention options that were prompted involved some kind of education. The CHW from Masike advised on giving education on family planning; ‘to make them have a family which they can take care of, so that we prevent malnutrition’. A man from Panyakoo suggested that people should be taught means of income generation to eradicate poverty and thereby financial constraints to food access. Interestingly, many interviewees recommended the provision of nutrition education as an effective additional intervention to improve the nutritional situation in the area. However, a man from Sota stated that with solely education, the food access barrier would still remain; ‘if you just give them education, ok they have the education, but they do not have enough money to buy’.

#### Target population

Discussions on gender equity in accessing nutrition interventions have been one topic that came up frequently during the interviews. Especially, when talking about the possible target population for nutrition interventions, opinions varied. Initially, some participants suggested that only women should receive nutrition education, because ‘women are the ones who are staying most of the time with the families and have to do the cooking, they should know of the important things which the children need’, as a 23-year-old man from Sota explained. But as the discussion continued, female interviewees agreed that according to their sociocultural context, they need their husband’s support and approval in case they want to adapt customs within the family, including dietary practices.

‘We should think of fathers or men, because they are the ones who go and get the money there. Because as a mother you can have the knowledge, but then you do not have the money to go and buy. If you ask, he is going to refuse. So, the men should be the ones to be taught’. (26-year-old woman from Sota)

To overcome this barrier, some participants suggested involving male ambassadors or role models from the community. A 31-year-old man from Masike explained that this could ‘make some other men feel like they can come and attend’. A few female participants highlighted that men would only come if they were rewarded for their attendance, or punished for being absent.

#### The possible role of Community Health Worker

Many participants suggested that CHW could play an important role in the implementation of nutrition interventions and the recruitment of motivated people from the community to take part in the interventions. The CHW showed great willingness to be involved within interventions targeting malnutrition in their communities; however, they explained that several requirements had to be met in order for the collaboration to succeed. All CHW agreed that they primarily need adequate training on nutrition themselves.

## Discussion

### Key findings

This qualitative study explored the perceptions and beliefs regarding maternal and child nutrition, with a focus on DBM, as well as barriers and facilitators in accessing nutritious food among men, women and CHW in Rorya district in rural Tanzania. Based on the results, the three most significant topics associated with malnutrition that were identified in this study include the large knowledge gaps concerning overweight and obesity as a health problem and the concept of DBM, changing weather patterns and its implications on food supply and the socio-cultural drivers including gender roles and household dynamics.

#### Knowledge on malnutrition

Participants had limited knowledge on the concept of DBM, adverse outcomes from obesity and overweight and other associated health impacts. This is in line with earlier studies among school children and their parents in Tanzania^(31)^. The limited knowledge on the DBM in the Rorya district could be attributed to a lack of educational possibilities, as well as to the influence of beliefs that overweight and obesity are signs of prestige and good life rather than a health problem^(32)^. Additionally, a lack of knowledge on the widespread nutritional transition that is occurring in many low- and middle-income countries, including Tanzania, further compounds the lack of understanding for the DBM. Nutritional transition refers to a shift from traditional dietary habits to a more high energy and low nutrient dense diet^(33)^. This entails the consumption of refined foods and cheap fats in combination with a more sedentary lifestyle and less physical activity^(34)^. Limited knowledge on childhood overweight and obesity has previously been associated with a decreased success rate of nutrition interventions tackling the DBM^(35)^. Previous studies in South Africa, Ghana and Burkina Faso have shown that education and knowledge are necessary, but are not on their own sufficient to support improvements in women’s and children’s nutritional status^(36)^. A recent review suggests that the combination of education interventions with other interventions such as food supplementation or conditional cash transfers might be more successful in improving nutritional status in infants^(37)^. The importance of nutritional education and counselling has also been highlighted by the 2021 Lancet Maternal and Child Undernutrition Series, in which nutrition counselling in the antenatal period was linked to improved dietary diversity, better adherence to micronutrient supplements and food security in pregnant women^(38)^.

#### Changing weather patterns

The impact of changing rain patterns has been mentioned repeatedly as one of the most important environmental factors to impact the quality as well as the quantity of the food available for community members in the Rorya district. Changing climates and seasonal fluctuations have been observed at an increasing frequency across SSA in the last couple of years^(14)^. This could result in an increased intensity of precipitation, resulting in flooding or more dry spells, which could affect food availability across SSA in the future^(39)^. Water supply for agriculture production in Tanzania is largely dependent on rainfall, thus deviation of traditionally consistent weather pattern of timely rainy seasons could influence crop yields and therefore impact access to nutrition in this region^(39)^.

With more unpredictable rain patterns, functioning irrigation systems could be one of the possible options to reduce further water stress on the farm land. In Tanzania, where smallholder farming provides over 70 % of the food supply, current developments are focused on strengthening smallholder irrigation systems^(40)^. Endeavours to increase knowledge on smart irrigation systems, using the close proximity to Lake Victoria, are currently underway in the catchment of Shirati. These systems, however, are both technical and institutional complex and often require national financing and policy programmes for its implementation^(40,41)^.

#### Economic and socio-cultural drivers

Participants stated that limited income and financial constraints remain a significant barrier in accessing nutritious food. Interventions aiming at creating new income-generating activities in countries with high rates of malnutrition have increased over the last years. Several projects have shown that improving the socio-economic status of families has a direct effect on maternal and child nutritional status^(42)^. In particular, interventions that aim at increasing the influence of women on nutrition-related decisions, such as involving women in problem-solving participatory groups, have shown to improve maternal and child health in other low-resource settings^(43,44)^.

Interestingly, both male and female participants have stated that both genders should be involved in the planning as well as in the implementation of future nutrition interventions in Rorya district.

Traditionally, men are considered the provider and the controller of the financial resources in many Tanzanian households. Whereas the purchase and preparation of food as well as child feeding is often regarded as sole responsibility of the women. While traditional gender roles prevail in many rural communities in East Africa, there has been a shift observed in men’s roles in nutrition over time, according to studies conducted in Malawi, Kenya and Uganda, where men are increasingly becoming involved in providing and purchasing food as well assisting with household-related tasks^(20,21)^.

Previous studies have shown that educating the wider family on nutritional-related topics, including men and mother in laws, can have a positive effect on infant nutritional outcomes^(21,45)^. This emphasises the importance of considering shifting gender roles and family dynamics in any future interventions aiming at tackling the DBM in this area.

### Recommendations

Based on our results and previous studies in rural Tanzania, there is a need for creating more awareness on the DBM and the health impact of overweight and obesity among the general population as well as health care workers in Rorya district. Therefore, fostering knowledge on the DBM among CHW could be a first step towards tackling the issue of DBM. Educational activities for CHW could be facilitated by the Shirati KMT Hospital, which has previously played a key role in providing education for CHW in the region. CHW in turn could deliver a variety of health education activities and counselling session in local health care facilities in the district as well as in local community meeting spaces. To improve sustainability of the intervention’s regular follow-up meetings, organisation of transport, phones for communication, a budget for teaching material and adequate supervision provided by either local government representatives or professionals who train the CHW are needed. The integration within already existing health care structures in the region and ownership of the local health care providers is essential for the success of any further intervention targeting the DBM.

### Strength and limitations

One of the strengths of the current study is its contribution to the current knowledge on perception and beliefs related to the double burden of malnutrition in rural Tanzania. Participants were local community members, including women, men and CHW of a range of ages and from different backgrounds in the region. A well-trained research team with an experienced local translator carried out the research and analysed the data using standardised methods.

Non-purposive sampling of participants can be seen as a limitation, as we only included people who attended the dispensary and who were willing to participate. As the interviews were translated into English, minimal loss of meaning from both questions and answers was inevitable. The absence of a second translator and logistical constraints to verify the interpretation of the participant’s responses by performing a member check, as well as the absence of a conceptual framework or model in the study is considered another limitation of this study.

### Conclusions

The DBM is a complex and multifaceted issue that is influenced by a variety of factors.

Environmental factors and the socio-cultural context play an essential role in the determinants for DBM, which underlines the importance of understanding the local context and the nutrition practices and beliefs of the respective communities. In order to address the various burdens of malnutrition in its full complexity in rural Tanzania, a more comprehensive approach is needed. CHW could play a key role in facilitating some of the suggested solutions, including health education activities for men and women and nutritional counselling and increasing awareness on the underlining causes of the double burden of malnutrition. The aim of future interventions should be to work towards a more inclusive approach, attempting to reframe nutrition as women’s responsibility, to avoid stigma of men who want to get involved and to facilitate comfort in addressing traditional female topics. Therefore, including men both in the set up and in the implementation of future nutrition interventions could offer valuable new possibilities in rural Tanzania.
